# The use of urinary fluoride excretion to facilitate monitoring fluoride intake: A systematic scoping review

**DOI:** 10.1371/journal.pone.0222260

**Published:** 2019-09-11

**Authors:** Oladipo S. Idowu, Liane B. Azevedo, Ruth A. Valentine, Josie Swan, Priyanka V. Vasantavada, Anne Maguire, Fatemeh V. Zohoori

**Affiliations:** 1 School of Health and Social Care, Teesside University, Middlesbrough Tees Valley, United Kingdom; 2 School of Dental Sciences, Newcastle University, Framlington Place, Newcastle Upon Tyne, United Kingdom; University of Mississippi Medical Center, UNITED STATES

## Abstract

**Background:**

As a recognised effective and economical agent for dental caries prevention, fluoride has been used in many different fluoridation schemes implemented across the world. Considering the narrow ‘dose-gap’ between the benefit of caries reduction and the risk of dental fluorosis, it is recommended that fluoride intake is monitored by measuring urinary fluoride excretion. The aim of this scoping review is to map the current literature/evidence on fluoride intake and excretion studies in relation to the study population, settings, type of study design, methodology, and analytical approach.

**Methods:**

Embase/Ovid, MEDLINE/Ovid, CINAHL/EBSCO, Scopus/Elsevier were searched for relevant articles until April 2018. Studies were included if they reported intake and excretion of fluoride in healthy humans of all age groups. Findings were explored using a narrative synthesis to summarise studies characteristics and outcome measures.

**Results:**

Removal of duplicates from the originally 2295 identified records yielded 1093 studies of which 206 articles were included. Only 21.6% of the studies were conducted in children (<8-year-olds). Most studies (38.8%) used drinking water concentration as a proxy for fluoride intake, whereas only 11.7% measured fluoride intake from all sources. Of the 72 studies that measured dietary fluoride intake, only 10 reported the validity of the employed dietary assessment method. Only 14 studies validated the urine sample collection methods. No information on the validity of the employed analytical method was reported by the majority (64.6%) of studies. Only a small proportion (8.7%) of the included studies investigated the association between fluoride intake and excretion.

**Conclusion:**

The findings reveal much variability in terms of conducting the studies and reporting the findings, illustrating a high heterogeneity in data collection across settings and populations. Future studies should provide more detail on sampling technique, measurement protocols (including validation), and on clearly defining the relationship between intake and urinary excretion of fluoride.

## Introduction

While dental caries is a largely preventable condition, it still remains an important global public health problem, affecting 60–90% of schoolchildren and the vast majority of adults [1]. The World Health Organisation (WHO) has officially endorsed the use of fluorides for population-based prevention of dental caries since the late 1960s [1]. The goals of community-based public health programmes are generally to provide regular, low-level exposure to fluoride in the community through appropriate means such as fluoridated water, salt, milk and fluoride toothpaste. Studies have shown that it can be quite challenging to attain effective fluoride-based caries prevention without the development of some degree of dental fluorosis, which can occur as a result of excessive ingestion of fluoride during tooth development. A key oral health promotion strategy is therefore to maximise caries reduction while minimising fluorosis. A fluoride intake of 0.05–0.07 mg/kg body weight (BW)/day in children <12 years of age has been suggested as optimum for caries prevention; whereas an intake of more than 0.1 mg/kg BW/day could increase the risk of occurrence of dental fluorosis [2]. Despite the widespread use of these guidelines in authoritative advisory recommendations for many years, the associated values and thresholds have been recently questioned due to their empirical origin [3–5]. Recent studies have also shown changes in the sources of ingested fluoride as well as in the prevalence and severity of dental fluorosis [3]. The Iowa Fluoride Study (IFS), which is the most comprehensive longitudinal cohort study on the association between fluoride intake, dental caries, and dental fluorosis, has also reported an overlap in mean fluoride intake between groups with dental caries and groups with fluorosis [6]. These reports indicate the need for research to clearly establish an optimum (or adequate) and upper limit of fluoride intake. The recent fluoride symposium on appropriateness of guidelines for fluoride intake [3] also highlighted the need for more nuanced guidelines on fluoride intake to help inform policy with the aim of introducing change in fluoride intake or leading to the adjustment of the timing of fluoride intake across the critical period of ages 0 to 6 years to reduce the risk of occurrence of fluorosis [4]. Assessment of total fluoride intake in the population is quite challenging due to exposure to multiple dietary and non-dietary sources of fluoride as well as the difficulty in measurement of the amount of unintentional swallowing of toothpaste by young children.

As urine is the most important metabolic pathway for elimination of fluoride from the body, it has been considered as a useful biomarker for contemporary fluoride exposure at a population- but not individual- level [7]. The relationships between intake and urinary excretion of fluoride have been examined for different age groups to establish the value of urine for prediction of fluoride intake according to age. A strong correlation between fluoride intake and urinary fluoride excretion has been reported for a pooled sample of children aged 7 years or younger as well as for adults aged 18 to 75 years [8]. However, no such correlation was found with a narrower age group of 6 to 7 year olds [9]; indicating the need for more fluoride intake and excretion studies in specific age groups. One of the conclusions of the recent fluoride symposium [3] was that “… *there is not enough evidence to use biomarkers of fluoride exposure such as urine and nails*, *despite the fact that they take into account the absorption rate*” along with the suggestion of more research being needed to establish a reliable biomarker of fluoride exposure [5].

Many factors can modify fluoride metabolism and consequently alter the relationship between fluoride intake and excretion, including age (skeletal growth), dose and form of fluoride as well as acid-base disturbances (e.g. altitude of residence), renal impairment, physical activity, nutritional status, composition of diet, and genetics [10]. Nevertheless, the implementation of fluoride interventions vary considerably across countries. For instance, community water fluoridation has currently been implemented in 25 countries and fluoridated salt is available in 23 countries [11]. Fluoridated milk is also given to school children in a few countries [12]. In addition, fluoridated toothpastes are widely used globally and many populations have naturally occurring fluoride in their drinking water supply. A mix of all these potential fluoride vehicles, with a complex fluoride availability, can influence fluoride excretion.

Fluoride intake and excretion studies also have the potential to reach erroneous conclusions if they use an unfocussed study design, invalid methods of data collection (particularly fluoride exposure data) or non-standardised analytical techniques. For example, a study in which the existing analytical techniques for fluoride analysis were examined amongst nine internationally recognised laboratories, showed inconsistencies in the use of these fluoride assay techniques [13]. The latter study also reported a statistically significantly difference in the average fluoride concentration of urine and beverage samples amongst the nine laboratories when using their own standard operating protocols and assay techniques. Future research should be able to add more nuanced understanding on fluoride intake in specific situations by ideally following standard operating protocols.

Direct assessment of total fluoride exposure in a population (particularly children) can be difficult and expensive. Measurement of fluoride levels in urine has been suggested as an alternative method for estimation of fluoride exposure in populations and consequently as a basis for decisions on the use of fluoride for caries prevention. However, a clearer perspective is needed to better understand the association between fluoride intake and urinary fluoride excretion and specific situations in which urinary fluoride excretion is strongly associated with intake. Therefore, the aim of this scoping review was to map the current literature/evidence on the association between fluoride intake and excretion. The specific objectives of this review were to map this association in relation to the study population, setting, type of study design and methodology, including the analytical approach used. The ultimate aim of this systematic scoping review is to review the observational data found in fluoride intake and excretion studies in humans to provide a basis for more targeted research questions to address any research gaps and inform the design of future interventions.

## Methods

This systematic scoping review was guided by the Joanna Briggs Institute for systematic scoping reviews [14–16]; and reported according to the Preferred Reporting of Items for Systematic Reviews and Meta- Analyses (PRISMA) Statement [17]. A detailed review protocol can be obtained from the Open Science Framework (DOI 10.17605/OSF.IO/9CQGE).

### Search strategy

A three-step approach was applied for the search [14–16]. The initial search was performed with MEDLINE (through Ovid) and CINAHL databases using the terms identified based on PEO (Population, Exposure, Outcome). A second search was then undertaken across four electronic databases using all identified reports and articles until April 30, 2018: Embase/Ovid (1974–2018), MEDLINE/Ovid (1946–2018), CINAHL/EBSCO (1997–2018), Scopus/Elsevier (1950–2018) (S1 File). No limits were set for years of publication on the database search. All citations were then imported into the web-based bibliographic manager Endnote X8, and duplicate citations were removed.

### Eligibility criteria

Searches were limited to studies in English and were included if they; (a) were conducted with heathy participants of any age group, gender or ethnicity; (b) were carried out at nursery, schools, preschool, kindergarten, child care centres, hospitals or community settings; (c) estimated fluoride intake from any customary sources of fluoride including water, diets, dental products and supplements and; (d) estimated urinary fluoride concentration or excretion.

Studies were excluded if they measured occupational fluoride exposure. Studies were also excluded if they were reviews, opinion papers, policy documents, case reports, editorials or letters.

### Process of study selection

A two-stage screening process was used to assess the relevant studies. The first level of screening involved only the study title and abstract by two reviewers (IO and JS). Afterwards, the full texts of the included studies were examined independently by two reviewers: IO and one of the co-authors (VZ, LA, RV, PV). Discrepancies were resolved by discussions and consensus or by consultation with a third reviewer (VZ or LA).

### Data charting process

A standardised data extraction tool was developed by the research team and included the following information: author(s); year; title; aim of study; study design; country; setting; number of participants; age; gender; intake and excretion data; methods of data collection, analytical procedures, and outcome(s). The developed data extraction tool was tested with 10% of the articles before implementation. Data extraction was then carried out by one reviewer (IO) and verified by another (PV). Due to the work undertaken being a scoping review, no quality appraisal was undertaken [16].

### Data synthesis

The extracted data were originally recorded in Excel and then imported to SPSS (version 22). Descriptive analysis was used to report the studies by their characteristics and outcome measures:

**A**. **Studies’ characteristics:**

- Year: which was divided into two groups according to the year of publication; “prior to-” and “at or after-” 1999. This was based on the year of release of the 1999 World Health Organisation (WHO) manual guidelines on monitoring fluoride excretion in community prevention programmes [18];
- Country: which was divided into low income economy, lower middle income economy, upper middle income economy and higher income economy, according to the World Bank Group classification [19];
- Setting: nursery/schools, hospitals, community or unclear;
- Age: 0–8 years, 9–16 years, 17 years and above, or a combination of age groups;
- Gender; male, female, or both;
- Study design (non-randomised controlled trial, randomised controlled trial, uncontrolled before and after studies, cross-sectional, or longitudinal studies.

**B**. **Outcome measures**

- Reporting of fluoride concentration in the area;
- Fluoride intake and sources: water, diet, dental products, supplements, or a specific combination of two or more sources—such as water and dental products, diet and supplement, diet and dental products, a combination of diet, supplement and dental products;
- Method of assessment of dietary fluoride intake: 24-h dietary recall, diet history, duplicate method, food diary, food frequency questionnaire (FFQ),semi-FFQ, household survey, observed food frequency, or not reported;
- Method of assessment of fluoride intake from dental products: sample collection–i.e. toothpaste applied/expectorate collected, toothbrushing questionnaire, or not reported;
- Fluoride excretion: urine, or urine and faeces;
- Assessment of fluoride in urine: urinary fluoride concentration, urinary fluoride excretion (by collecting 24-h urine, or spot urine, or time controlled urine), or not specified.
- Analytical method: fluoride-ion selective electrode, gas chromatography, spectrophotometry, titration, ion-exchange chromatography, or not reported;
- Validity of data and methods: fluoride intake, fluoride excretion (urine collection) and fluoride analytical method, or not reported;
- Reporting of the investigation of any relationship between fluoride intake and fluoride excretion.

Findings were then further explored using a narrative synthesis.

## Results

### Studies’ characteristics

**Fig 1:**
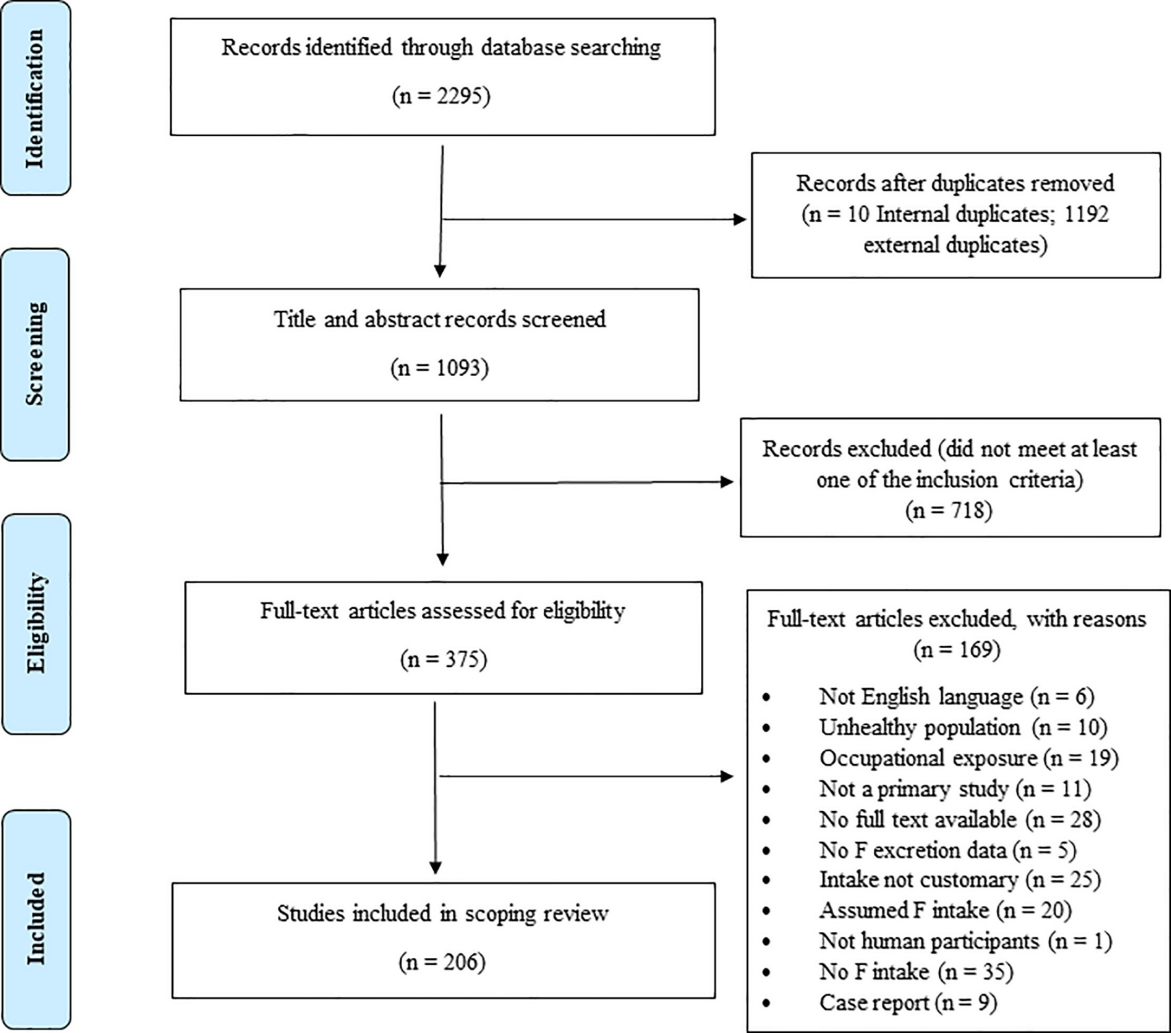
Flowchart outlining identification of papers for inclusion following the Prisma flow diagram.

The search of four databases yielded 2295 records (Fig 1): Ovid Medline, 613; Ovid Embase, 763; Scopus, 867; CINAHL, 52. After removal of duplicates, the titles and abstracts of 1093 studies were screened for eligibility and 718 studies were excluded. The full text of 375 potentially relevant articles were further screened for eligibility. A total of 169 studies were excluded for different reasons (Fig 1) and 206 studies met the inclusion criteria described above.

**Fig 2:**
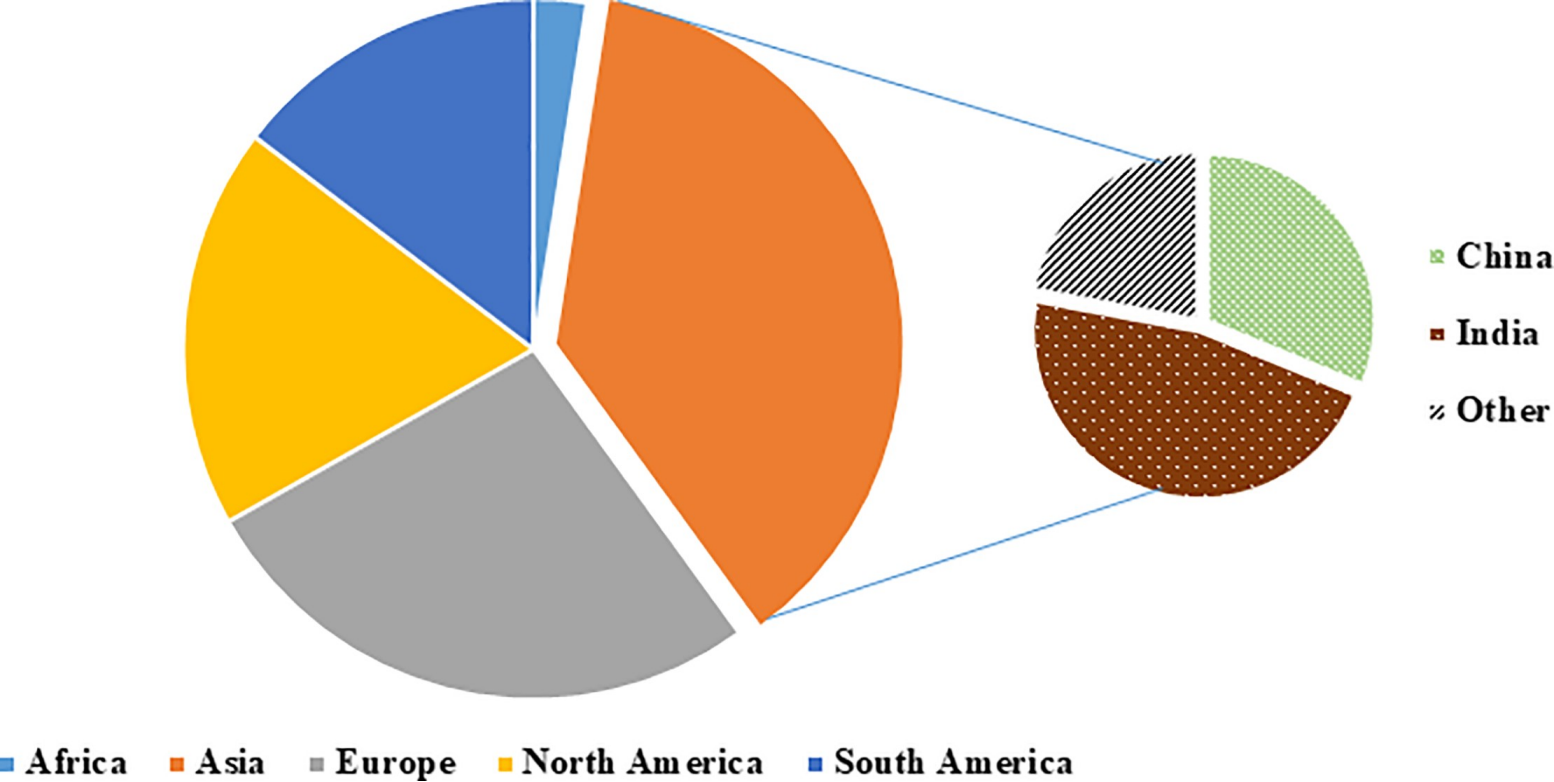
Distribution of included studies according to continent.

Studies originated from 43 countries across most continents, with the majority from India (n = 36), USA (n = 35) and China (n = 24) (Fig 2). Table 1 presents the characteristics of the included studies. A higher proportion (57%) of the studies was published after 1999: on average, five papers were published per year between 1999 and 2018 compared to two papers per year in the years up to 1999. The vast majority of the studies (79.1%) were conducted in either higher- or upper-middle- income economy countries and the predominant setting was at community (56.3%). More studies were conducted in adults (30.4%) or combining different age groups (39.2%) and predominately on both genders (82.6%). While a range of study designs were applied, the majority used a cross-sectional design (64.6%).

**Table 1:** Studies’ (n = 206) characteristics.

Category	Frequency (percentage)
Year of publication	
1949–1998	89 (43.2)
1999–2018	117 (56.8)
Country	
Low Income Economy	4 (1.9)
Lower Middle Income Economy	39 (18.9)
Upper Middle Income Economy	56 (27.2)
Higher Income Economy	107 (51.9)
Study setting	
Community	116 (56.3)
Hospital	30 (14.6)
Nursery/School	52 (25.2)
Unclear	8 (3.9)
Age group categories	
0–8 years	44 (21.6)
9–16 years	18 (8.8)
17 years and above	62 (30.4)
Any of above combinations	80 (39.2)
Gender	
Male	26 (12.6)
Female	12 (5.8)
Both	168 (81.6)
Study design	
Randomised controlled trial	9 (4.4)
Non-randomised controlled trial	18 (8.7)
Before and after (Intervention)	44 (21.4)
Cross-sectional studies	133 (64.6)
Longitudinal study	2 (1.0)

### Outcome measures

Tables 2–4 show the assessed variables and employed methodology of the included studies.

**Table 2:** Outcome measures related to fluoride intake, specified in the included studies (n = 206).

	No of studies (percent)
Was the water fluoride concentration of the area reported?	
No	113 (53.9)
Yes	93 (45.1)
Sources of intake	
Proxy (water)	80 (38.8)
Water and dental products	4 (1.9)
Water and supplement	3 (1.5)
Diet	51 (24.8)
Supplements	8 (3.9)
Dental products	7 (3.4)
Supplement and dental product	1 (0.5)
Diet and supplement	28 (13.6)
All sources	24 (11.7)
Dietary intake assessment method	
24-h dietary recall	6 (2.9)
Diet history	2 (1.0)
Duplicate method	16 (7.8)
Food diary	17 (8.3)
Food Frequency Questionnaire	16 (7.8)
Household survey	2 (1.0)
Observed food frequency	13 (6.3)
Not reported	134 (65.0)
Oral hygiene assessment method	
Toothpaste applied/expectorated toothpaste and mouth rinses	16 (7.8)
Questionnaire	20 (9.7)
Not reported	170 (82.5)
Validity of dietary intake assessment methods	10 (13.9*)

* Out of the 72 studies that measured dietary fluoride intake

**Table 3:** Outcome measures related to fluoride excretion (n = 206 studies).

	No of studies (percent)
Excretion method	
Urine alone	183 (88.8)
Faeces and urine	23 (11.2)
Method of assessment of urinary fluoride excretion	
24-h urine	72 (35.0)
Spot urine	71 (34.5)
Time controlled urine	22 (10.7)
Not specified	41 (19.9)
Method of fluoride analysis	
Fluoride Ion-selective-electrode	183 (88.8)
Gas chromatography	2 (1.0)
Spectrophotometry	3 (1.5)
Titration	2 (1.0)
Ion-exchange chromatography	1 (0.5)
Not reported	15 (7.3)
Validity of testing of methods	
Urine collection method (24-hour collection)	13 (18.1)
Method of validity testing:	
- Urinary flow rate	3 (2.1*)
- Urine volume	1 (7.7*)
- Creatinine	5 (38.5*)
- Combinations of any of above methods	4 (30.8*)
Fluoride analysis methods	
Reported	73 (35.4)
Not reported	133 (64.6)

* Out of the 13 studies that measured validity of urine collection

**Table 4:** Studies which explored relationships between fluoride intake and excretion data.

Study outcome	No of studies*	Reference
Association between fluoride intake (dietary and non-dietary) and fluoride excretion	18	9, 23, 26–39
Association between fluoride concentration of water and fluoride excretion	28	27, 40–66
Fractional urinary fluoride excretion	16	9, 28, 31–32, 67–78
Fluoride balance	15	38, 79–93
Bioavailability	7	29, 94–99
Fluoride retention	9	8, 27, 37, 100–105

* Some studies explored more than one relationship.

A considerable number of studies (n = 113; 53.9%) included in the review did not report the water fluoride concentration of the area investigated (Table 2). Most studies used water as a proxy for fluoride intake, while only 24 (11.7%) studies measured fluoride intake from all sources. In the 72 studies that measured dietary fluoride intake, the most frequently used assessment methods were duplicate method (n = 16), food diary (n = 17) and food frequency questionnaire (n = 16). Only 10 studies (out of the 72) reported the validity of the employed dietary assessment method: duplicate method (n = 5), 24-hour dietary recall (n = 3) and food dietary assessment method (n = 2). Only 36 (27.5%) studies measured fluoride intake from ingestion of dental products of which 20 (55.5%) studies used a questionnaire as the assessment tool.

A small proportion (n = 23; 11.2%) of studies measured fluoride excretion from both urine and faeces (Table 3), whereas the majority (n = 183; 88.8%) assessed fluoride excretion from urine alone. Almost one-fifth of the studies did not specify the method of urine collection and, when reported, most used either a 24-hour (35%) or spot urine (34.5%) collection method. Out of the 72 studies in which 24-hour urine collection was collected, only 13 (18.1%) studies validated the sample collection either by urinary flow rate (n = 3), urinary volume (n = 1), creatinine ratio (n = 5) or any combination (n = 4). Most of the included studies (88.8%) measured urinary fluoride concentration using a fluoride ion selective electrode (F-ISE). However, no information on the validity and reliability of the employed analytical method was reported by the majority of the studies (n = 133; 64.6%).

Less than half (45%; n = 93) of the included studies reported the investigation of any relationship between fluoride intake and excretion. Of the studies [8, 9, 20–100] that investigated the intake/excretion relationship, the majority (30%; n = 28) reported the association between fluoride concentration of water and fluoride excretion, whereas only 18 studies (19%) reported the association between fluoride intake and fluoride excretion (Table 4).

## Discussion

This systematic scoping review synthesised the evidence for the association between fluoride intake and fluoride excretion in healthy human participants including the characteristics of the studies and findings of the existing research.

The review showed that number of published studies increased post 1999; which might be due to a publication by the World Health Organisation (WHO) on “Monitoring of renal fluoride excretion in community prevention programmes in oral health” [18] in 1999. In that WHO guideline only 11 fluoride intake and/or excretion studies were cited and this may have led to a re-attraction of the attention of researchers around the world to the paucity of data, stimulating research on the suitability of urine for monitoring fluoride exposure. As a result, the latest WHO guidance [7], published in 2014, cited more than 34 studies on fluoride intake/excretion in children. However, the main conclusion of the 2014 WHO guidance on assessment of renal fluoride excretion was mainly based on a study [8] in which the ability of 24-hour urine sample to predict fluoride exposure was investigated by examining the 17 published reports of the simultaneous measurement of fluoride intake and excretion for 212 pooled samples of children aged less than 7 years (taken from 9 studies in children) and for 283 adults aged 18–75 years (taken from 8 studies in adults) who consumed ‘westernised’ diets.

The findings of this scoping review show an unbalanced geographical distribution of articles and heavy concentration of studies from higher-income-economy countries. Interestingly, many studies were conducted in Asia, particularly in China and India. This could be related to; i) an increase in budgets for research and development [101] and/or; ii) a need for research and monitoring of fluoride exposure due to the extent of fluoride toxicity from contaminated water and polluted air, in those countries.

Studies suggest that the first two years of life are the most important period for development of fluorosis in early-erupting teeth, whereas the first eight years of life appear to be the most important period for development of dental fluorosis in late-erupting permanent teeth [102]. However, the findings of this review show that of the 206 included studies, only 44 (21.6%) were conducted in children younger than 8 years old. The lower number of studies involving children could be due to the various challenges (such as ethical conduct as well as cooperation of children and their parents) and costs of conducting paediatric research. However, more studies involving children are essential as research with adults in not easily generalized or extrapolated to infants and young children due to their anatomical and physiological differences.

The findings also reveal great variability in terms of study conduct and the reporting of findings, illustrating a high heterogeneity in data collection across settings and populations. One of the main sources of heterogeneity was the methods of assessment of fluoride intake (from diet and toothpaste ingestion). In particular, the methods of dietary data collection widely differed between the studies (Table 2), emphasizing the need for the development of a gold standard method for more accurate estimation of the systemic ingestion of fluoride from diet. Furthermore, few studies reported detailed information on the validity of dietary data collection (Table 3). Since gastric absorption and renal excretion of fluoride are pH-dependent, any diet-induced changes in the pH of the stomach as well as urinary pH could decrease or increase the concentration of fluoride in urine. For example; i) the bioavailability of fluoride from water is almost 100%, whereas a mixed diet may reduce absorption of fluoride by 47% [103] and; ii) a vegetarian-based diet increases fluoride excretion. To establish an exact association between fluoride intake and excretion, the type of diet should, therefore, be evaluated and reported accurately.

This scoping review also shows the heterogeneous nature of the methods used to collect urine samples (Table 3). When spot urine samples were collected, few studies provided detailed information on the time of sample collection (e.g. fasting or non-fasting; before/after meal/brushing, etc). The fluoride concentration of a spot urine sample can be influenced by factors such as hydration status, time of collection (in relation to time of fluoride ingestion) and length of accumulation of urine (and fluoride) in the bladder. A single spot urine sample, collected after overnight fasting, could be a potentially useful indicator of chronic fluoride exposure for water fluoridation schemes, in which the community ingests a constant low level of fluoride throughout the day. However, when monitoring inadvertent F ingestion, such as through swallowed toothpaste, or through community-based milk fluoridation programmes when participants receive a single fluoride dose at a particular time of day, it is important that more spot urine samples are collected to allow the most appropriate and accurate evaluation of the programme. Although creatinine-adjustment methods have been reported [20, 104] as an appropriate method for estimation of urine fluoride concentration of spot urine samples, and consequently urinary fluoride excretion, they have not been widely explored with different populations and intakes (i.e. different age groups, diet, etc). Likewise, although the majority of the included studies measured fluoride concentration of samples using a fluoride ion-selective-electrode, the validity of the method used was reported in very few studies (Table 3) and this information is essential to accurately compare and understand the findings of the various studies.

Any relationship between fluoride intake and urinary fluoride excretion was explored in only 45% of the studies. The association between fluoride concentration of water and fluoride excretion was reported in 28 studies, whereas only 18 studies (9%) reported the association between fluoride intake and fluoride excretion (Table 4). Considering the multiple and increasing sources of fluoride exposure, and the rise in the “halo effect” of fluoride through globalisation of the food and drink industry [105], the relevance of studies in which fluoride concentration of water is used as a proxy for fluoride intake could be questionable. In addition, due to the diversity of the methods of data collection and analysis, geographical locations and age groups, as well as the small number of studies (n = 18) in which the relationship between fluoride intake and fluoride excretion was investigated, no firm conclusion on the appropriateness of urine as a biomarker for fluoride exposure for all age groups and all dietary/oral hygiene habits can be drawn.

The main limitations of this review were that; i) the searches were limited to studies published in English which may lead to language bias and the omission of relevant reports published in other languages, ii) the “grey literature”, which could reduce the impact of publication bias, was not included (mainly due to the concern with the manageability of overwhelming volume of identified peer-reviewed articles) and; iii) being a scoping review, the quality of the included studies was not assessed and the conclusion was mainly based on the existence of studies rather than their quality.

## Conclusions

The diversity of the included studies in this review and their findings highlight the need for strong evidence (collected via reliable reproducible methods) on appropriateness of urinary fluoride as a suitable measure of fluoride exposure for different situations; e.g. different age groups and types of fluoride-based caries-prevention programmes across diverse communities with their different geographical locations and wide ranges of dietary and oral hygiene habits.

### Future studies

Future research should focus on: i) development (and validation) of gold standard universal methods for assessing fluoride intake from diet and toothpaste ingestion according to age group and dietary/oral hygiene habits; ii) development (and validation) of alternative methods for 24-hour urine collection such as urinary fluoride: creatinine ratio and urinary fluoride: specific gravity ratio in spot urine samples and iii) assessment of the ability of spot urine sample(s) to predict fluoride intake, according to age group, sources of fluoride exposure including fluoridation scheme.

Likewise, future studies should fully report detail on sampling technique (e.g. fluoride exposure, fluoride excretion), measurement protocols (including validation of the employed methodology), and clearly defined outcomes. Studies should also include reporting on the investigation of the relationship between total fluoride intake and urinary excretion of fluoride. Furthermore, researchers should ensure that potential differences between their findings and other studies/situations are included in their reporting.

## Supporting information

S1 File Search history conducted in four electronic databases. (PDF)

S2 File PRISMA-ScR Fillable checklist. (PDF)
