# Supplementary material for: The use of urinary fluoride excretion to facilitate monitoring fluoride intake: A systematic scoping review
Source: PLoS One. 2019 Sep 11;14(9):e0222260. doi: 10.1371/journal.pone.0222260 (PMC6738609; doi:10.1371/journal.pone.0222260)
Supplement: S1 File — (PDF) [file pone.0222260.s001.pdf]

S1 file: Search history conducted in four electronic databases

Database	CINAHL
Search query	S1 groundwater or consumption or dose* or intake or ingest* or expos* or (fluorid* N4 content) or fluoridat* Search modes - Boolean/Phrase<br>S2 (urin* OR renal OR excret*) N6 fluorid* Search modes - Boolean/Phrase<br>S3 ((urin* OR renal OR excret*) N6 fluorid*) AND (S1 AND S2) Search modes - Boolean/Phrase<br>S4 "(diet* or supplement* or dentifrice* or tablet or salt or milk or dental product* or fluoride varnish* or mouth rinse* or infant milk formula or food* or beverage or fluoridated water* or drink*)" Search modes - Boolean/Phrase<br>S5 "(diet* or supplement* or dentifrice* or tablet or salt or milk or dental product* or fluoride varnish* or mouth rinse* or infant milk formula or food* or beverage or fluoridated water* or drink*)" Search modes - SmartText Searching<br>S6 groundwater or consumption or dose* or intake or ingest* or expos* or (fluorid* N4 content) or fluoridat* Search modes - Boolean/Phrase<br>S7 (urin* OR renal OR excret*) N6 fluorid* Search modes - Boolean/Phrase<br>S8 ((urin* OR renal OR excret*) N6 fluorid*) AND (S6 AND S7) Search modes - Boolean/Phrase<br>S9 (diet* or supplement* or dentifrice* or tablet or salt or milk or dental product* or fluoride varnish* or mouth rinse* or infant milk formula or food* or beverage or fluoridated water* or drink*) Search modes - Boolean/Phrase<br>S10 S1 OR S9 Search modes - Boolean/Phrase<br>S11 ((urine* fluoride concentration* or urine* fluoride excretion or urin* fluoride level* or urin* fluoride retention or renal fluoride retention or urin* fluoride or urin* fluoride content or fluoride balance*)) OR ((urin* OR renal OR excret*) N6 fluorid*) Search modes - Boolean/Phrase

	S12 S10 AND S11 Search modes - Boolean/Phrase
Number of hits	52

Database	Medline
Search query	1. fluoride intake or fluoride ingestion or fluoride dose or fluoride exposure or fluoride content or fluorida*<br>2. exp Fluorides/ur [Urine]<br>3. 2 or urin* fluoride concentration* or urin* fluoride excretion or urin* fluoride level* or urin* fluoride retention or renal fluoride excretion or urin* fluoride or urin* fluoride monitor* or urin* fluoride content or fluoride balance*<br>4. 1 and 3<br>5. limit 4 to animals<br>6. limit 5 to humans<br>7. 4 not (5 not 6)<br>8. ((renal* adj4 fluorid*) or (excret* adj4 fluorid*)).mp. [mp=title, abstract, original title, name of substance word, subject heading word, keyword heading word, protocol supplementary concept word, rare disease supplementary concept word, unique identifier, synonyms]<br>9. (urin* adj6 fluorid*).mp. [mp=title, abstract, original title, name of substance word, subject heading word, keyword heading word, protocol supplementary concept word, rare disease supplementary concept word, unique identifier, synonyms]<br>10. 3 or 9 or 8<br>11. limit 10 to animals<br>12. limit 10 to humans<br>13. 10 not (11 not 12)

	14. (groundwater or consumption or dose* or intake or ingest* or expos* or (fluorid* adj4 content) or fluoridat*).mp. [mp=title, abstract, original title, name of substance word, subject heading word, keyword heading word, protocol supplementary concept word, rare disease supplementary concept word, unique identifier, synonyms]<br>15. exp Fluorides/ad [Administration & Dosage]<br>16. exp Groundwater/<br>17. drinking water/ or exp mineral waters/<br>18. water.mp.<br>19. exp Water Supply/<br>20. 14 or 15 or 16 or 17 or 18 or 19<br>21. 13 and 20<br>22. (diet* or supplement* or dentifrice* or tablet or salt or milk or dental product* or fluoride varnish* or mouth rinse* or infant milk formula or food* or beverage or fluoridated water* or drink*).mp. [mp=title, abstract, original title, name of substance word, subject heading word, keyword heading word, protocol supplementary concept word, rare disease supplementary concept word, unique identifier, synonyms]<br>23. 13 and 22
Number of hits	613

Database	EMBASE (Ovid)
Search query	1. (urine* fluoride concentration* or urine* fluoride excretion or urin* fluoride level* or urin* fluoride retention or renal fluoride retention or urin* fluoride or urin* fluoride content or fluoride balance*).mp. [mp=title, abstract, heading word, drug trade name, original title, device manufacturer, drug manufacturer, device trade name, keyword, floating subheading word, candidate term word]<br>2. ((renal* adj4 fluorid*) or (excret* adj4 fluorid*) or (urin* adj6 fluorid*)).mp. [mp=title, abstract, heading word, drug trade name, original title, device manufacturer, drug manufacturer, device trade name, keyword, floating subheading word, candidate term word]<br>3. 1 or 2<br>4. limit 3 to animals<br>5. limit 3 to human<br>6. 3 not (4 not 5)<br>7. (drinking water or mineral water or water supply or groundwater or consumption or dose* or intake or ingest* or expos* or (fluorid* adj4 content) or fluoridat*).mp. [mp=title, abstract, heading word, drug trade name, original title, device manufacturer, drug manufacturer, device trade name, keyword, floating subheading word, candidate term word]<br>8. (diet* or supplement* or dentifrice* or tablet or salt or milk or dental product* or fluoride varnish* or mouth rinse* or infant milk formula or food* or beverage or fluoridated water* or drink*).mp. [mp=title, abstract, heading word, drug trade name, original title, device manufacturer, drug manufacturer, device trade name, keyword, floating subheading word, candidate term word]<br>9. exp fluoride/ad, do, ih, im, ip, tr, tu, iv, po, pa, sb, li, tp [Drug Administration, Drug Dose, Inhalational Drug Administration, Intramuscular Drug Administration, Intraperitoneal Drug Administration, Intratracheal Drug

	Administration, Intratumoral Drug Administration, Intravenous Drug Administration, Oral Drug Administration, Parenteral Drug Administration, Sublabial Drug Administration, Sublingual Drug Administration, Topical Drug Administration]<br>10. 7 or 8 or 9<br>11. 6 and 10
Number of hits	763

Database	SCOPUS
Search query	((TITLE-ABS-KEY ((urin* OR renal OR excret*) W/6 fluorid*))<br>AND<br>(TITLE-ABS-KEY (water OR groundwater OR consumption OR dose* OR intake OR ingest* OR expos* OR (fluorid* W/4 content) OR fluoridat*)))<br>AND NOT<br>(TITLE-ABS-KEY ((animal* OR rats OR cattle) AND NOT (human* OR adult* OR child*)))
Number of hits	867
